# Applicability of Preoperative Nuclear Morphometry to Evaluating Risk for Cervical Lymph Node Metastasis in Oral Squamous Cell Carcinoma

**DOI:** 10.1371/journal.pone.0116452

**Published:** 2014-12-30

**Authors:** Masaaki Karino, Eiji Nakatani, Katsumi Hideshima, Yoshiki Nariai, Kohji Tsunematsu, Koichiro Ohira, Takahiro Kanno, Izumi Asahina, Tatsuo Kagimura, Joji Sekine

**Affiliations:** 1 Department of Oral and Maxillofacial Surgery, Shimane University Faculty of Medicine, Izumo, Japan; 2 Translational Research Informatics Center, Kobe, Japan; 3 Department of Regenerative Oral Surgery, Nagasaki University Graduate School of Medical Science, Nagasaki, Japan; University of North Carolina School of Medicine, United States of America

## Abstract

**Background:**

We previously reported the utility of preoperative nuclear morphometry for evaluating risk for cervical lymph node metastases in tongue squamous cell carcinoma. The risk for lymph node metastasis in oral squamous cell carcinoma, however, is known to differ depending on the anatomical site of the primary tumor, such as the tongue, gingiva, mouth floor, and buccal mucosa. In this study, we evaluated the applicability of this morphometric technique to evaluating the risk for cervical lymph node metastasis in oral squamous cell carcinoma.

**Methods:**

A digital image system was used to measure the mean nuclear area, mean nuclear perimeter, nuclear circular rate, ratio of nuclear length to width (aspect ratio), and nuclear area coefficient of variation (NACV). Relationships between these parameters and nodal status were evaluated by t-test and logistic regression analysis.

**Results:**

Eighty-eight cases of squamous cell carcinoma (52 of the tongue, 25 of the gingiva, 4 of the buccal mucosa, and 7 of the mouth floor) were included: 46 with positive node classification and 42 with negative node classification. Nuclear area and perimeter were significantly larger in node-positive cases than in node-negative cases; however, there were no significant differences in circular rate, aspect ratio, or NACV. We derived two risk models based on the results of multivariate analysis: Model 1, which identified age and mean nuclear area and Model 2, which identified age and mean nuclear perimeter. It should be noted that primary tumor site was not associated the pN-positive status. There were no significant differences in pathological nodal status by aspect ratio, NACV, or primary tumor site.

**Conclusion:**

Our method of preoperative nuclear morphometry may contribute valuable information to evaluations of the risk for lymph node metastasis in oral squamous cell carcinoma.

## Introduction

Lymph node metastasis strongly influences the five-year survival rate and prognosis of oral cancer. Besides treating the primary lesion, appropriate management of the cervical lymph nodes is an important part of oral cancer therapy [1]–[5]. Up to 30% of patients with a clinical N0 neck may still harbor occult metastasis [7], and how this can be best managed remains unclear [5], especially in cases that were clinically or radiographically diagnosed as lymph-node positive but no metastasis had been found in neck dissection [8], [9].

Lymph node metastasis is a complex multi-step process and cannot be explained by enlargement only [10]. Radical neck dissection has been considered the standard treatment procedure for cervical lymph node metastasis in oral cancer since Crile first described it in 1906 [11]. However, there has long been controversy over the indications, timing, and methods of neck dissection [2]–[5]. A reliable and accurate means of preoperative evaluation of cervical lymph node metastasis is therefore crucial for the correct management of oral cancer [1], [4], [6], [8], [12] and risk criteria should be established.

Many studies have reported the risk factors for cervical lymph node metastasis in oral cancer [8], [13]–[19]. Most have used a combination of several qualitative methods, including histopathologic parameters [13]–[16], gene expression [17]–[19], sentinel lymph node detection [20]–[24], and imaging techniques [25]–[28]. Quantitative analysis of nuclear variations has been undertaken for other types of lesions such as thyroid and breast cancers [29], [30]. In a previous study investigating the relationship between nuclear variations of squamous cell carcinoma of the tongue and cervical lymph node metastasis, we performed preoperative nuclear morphometry and found that this quantitative characterization the nuclear features of the primary tumor was a potential criterion for predicting lymph node metastasis in squamous cell carcinoma of the tongue specifically [8]. However, the potential risk for lymph node metastasis in oral squamous cell carcinoma (OSCC) differs depending on the anatomical site of the primary tumor, such as the tongue, gingiva, mouth floor, and buccal mucosa [12], [31], [32].

In this study, we performed preoperative nuclear morphometry for OSCC cells obtained on preoperative biopsy from primary tumors in the tongue, gingiva, buccal mucosa, and mouth floor. The investigated parameters were the mean nuclear area, mean perimeter, nuclear circular rate, ratio of nuclear length to width (aspect ratio), and nuclear area coefficient of variation (NACV) as an objective parameter of anisonucleosis. The relationships between these parameters and pathologic nodal classification (pN) status, including level of the metastatic lymph nodes, were evaluated retrospectively.

## Materials and Methods

### Data collection

Data were retrospectively collected for patients who were histopathologically diagnosed with OSCC and underwent surgical management including neck dissection at the Department of Oral and Maxillofacial Surgery, Nagasaki University Medical and Dental Hospital between January 1986 and January 2001 and the Department of Oral and Maxillofacial Surgery, Shimane University Faculty of Medicine between 1981 and 2012. Recurrent cases were excluded.

### Biopsy specimens and pathologic nodal classification

Biopsy was performed in all patients preoperatively and/or prior to neoadjuvant therapy. The biopsy specimens were fixed with 10% neutral buffered formalin for 24 h and were processed for routine paraffin embedded sections, then stained with hematoxylin and eosin.

**Figure 1:**
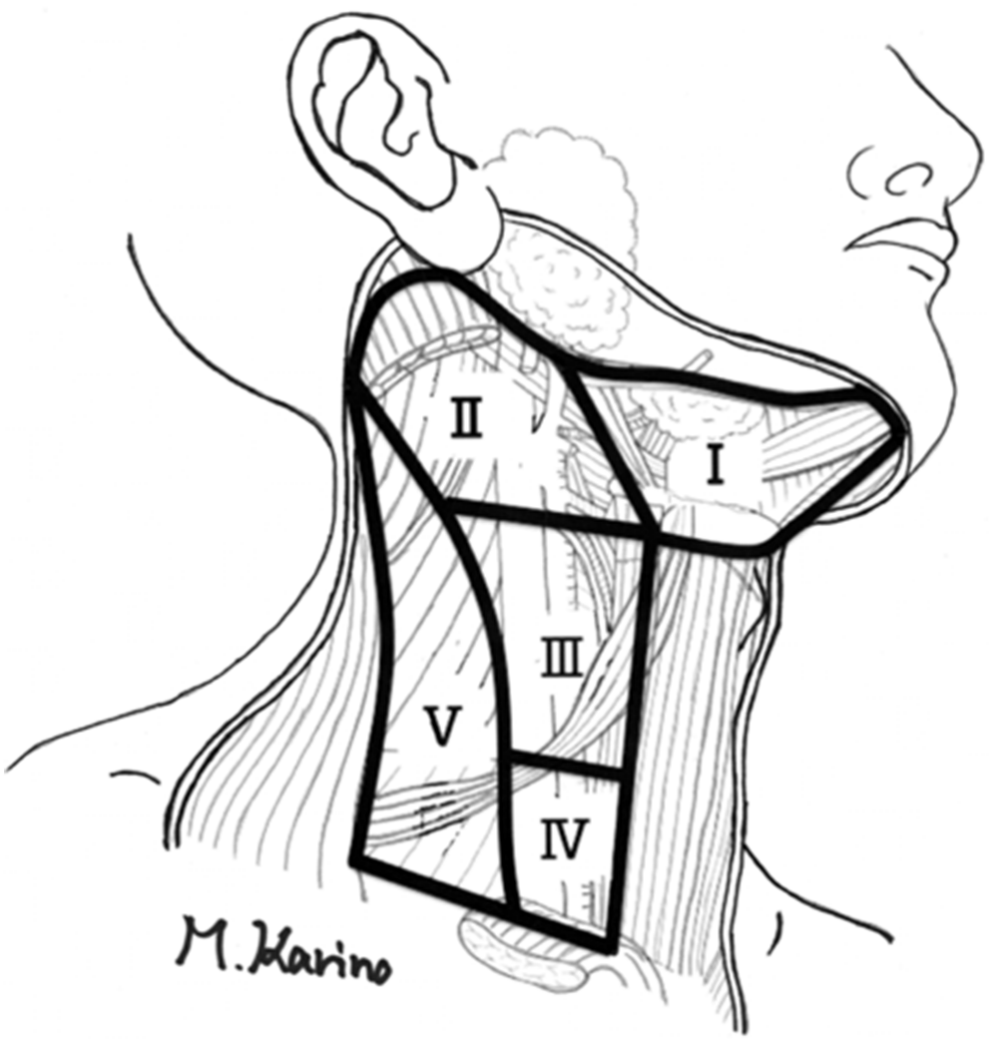
Illustration of each level of the cervical lymph node.

All the lymph nodes dissected from the biopsy specimens were examined for pN status and level of the metastatic lymph nodes. Cervical lymph node level was determined based on the cervical lymph node metastatic guide [33], as shown in Fig. 1.

### Image analysis and nuclear parameter measurements

Images of each section were stored using a standard light microscope (using x10 objective lens) connected to a computerized digital camera. The image data were analyzed by Mac Scope software (Mitani Co., Fukui, Japan) to estimate the various quantitative nuclear features (at least 100 nuclei per case). Nuclear margins were digitally marked under high power view on the computer screen to ensure measurement accuracy [8].

**Figure 2:**
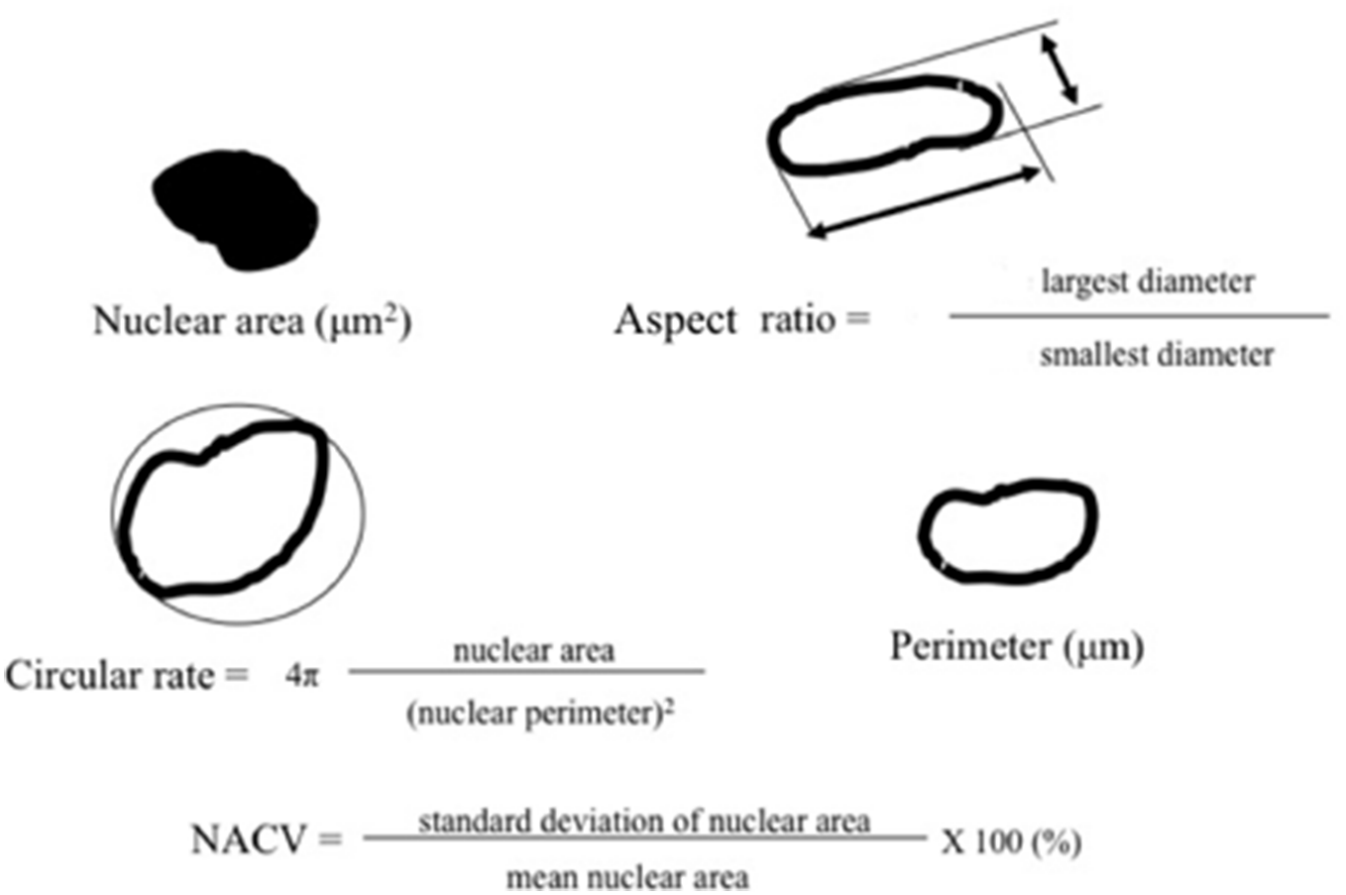
Illustration and formulas of parameters for quantitative estimation of nuclear parameters. NACV: nuclear area coefficient of variation.

Mean (standard deviation) values of the nuclear area and perimeter were calculated from counts of the pixels capturing the nuclei and their edges. The nuclear circular rate and aspect ratio were automatically calculated to determine variations in shape; briefly, in a round circle, the circular rate and aspect ratio values correspond to 1: if the object is elliptical, the circular rate is <1 and the aspect ratio is >1. NACV was calculated to express variations in size in individual cases (Fig. 2).

### Statistical analysis

To examine differences in patient characteristics between pN-positive and pN-negative patients, we performed *t*-tests for continuous variables. *p* values less than 0.05 were considered statistically significant.

Logistic regression analysis was performed to identify the risk factors for node-positive status. Odds ratios and confidence intervals (based on the Wald test) were also calculated. Candidate risk factors with *p* values less than 0.1 on the Wald test were selected. From among them, risk factors were determined by variable selection using Akaike’s Information Criterion. The optimal cutoff values for measurements were obtained with the minimum *p* values from the *t*-tests. All analyses were performed using SAS version 9.3 software (SAS Institute Inc., Cary, NC).

### Ethics statement

The Department of Oral and Maxillofacial Surgery, Shimane University Faculty of Medicine was responsible for the biopsy specimens used in this study. The Ethics Committee of Shimane University approved the study (Approval No.: 1286). Patients provided written informed consent for their data to be used in this study.

## Results

### Patient characteristics

The characteristics of 88 patients from whom data were collected are shown in Table 1. Patient age ranged from 35 to 84 years (mean, 64.4 years). The OSCC tumor sites were tongue (52 patients), upper gingiva (14 patients), lower gingiva (11 patients), buccal mucosa (4 patients), and floor of the mouth (7 patients). Of the 88 patients, 76 received neoadjuvant chemotherapy including pepleomycin sulfate (total volume 35–60 mg) or cisplatin (60–100 mg/m^2^), radiotherapy (30–40 Gy), or both chemo- and radiotherapy. In total, 45 patients underwent radical neck dissection, 38 underwent supraomohyoid neck dissection, 3 underwent biopsy of the submandibular lymph nodes (B), and 2 underwent functional neck dissection.

**Table 1:** Clinical and quantitative morphometric data of patients with primary OSCC.

Case	Age	Sex	Site	Differentiation	NAT	T	N	M	ND	pN	No oF pN	Level	Nuclear area	Perimeter	Circular rate	aspect ratio	NACV
1	65	M	tongue	well	C	2	0	0	Biopsy	0	0	-	79.1	32.2	0.869	1.42	29.2
2	61	M	tongue	well	C	2	0	0	Biopsy	0	0	-	54.6	26.4	0.874	1.37	30.6
3	82	F	tongue	well	C	2	0	0	Biopsy	0	0	-	73.7	30.9	0.857	1.43	40.6
4	35	M	tongue	well	C	1	0	0	RND	0	0	-	70.3	30.0	0.875	1.38	33.9
5	70	M	tongue	well	C	4	2b	0	RND	0	0	-	78.0	31.9	0.861	1.46	33.5
6	57	M	tongue	well	C	3	0	0	SOHND	0	0	-	48.0	24.8	0.843	1.48	45.0
7	78	F	tongue	well	C	2	0	0	SOHND	0	0	-	61.3	27.7	0.888	1.29	33.1
8	80	F	tongue	well	C	2	2b	0	SOHND	0	0	-	85.0	32.7	0.892	1.27	42.2
9	74	M	tongue	well	C	1	1	0	FND	0	0	-	51.6	25.6	0.871	1.39	32.8
10	64	M	tongue	well	C	4	2c	0	RND	0	0	-	50.6	25.4	0.862	1.43	36.6
11	52	M	tongue	well	C	1	0	0	FND	0	0	-	76.2	30.7	0.893	1.30	46.1
12	56	M	tongue	well	C	2	0	0	SMND	0	0	-	72.8	31.0	0.845	1.46	36.1
13	69	M	tongue	well	C	3	2b	0	SOHND	0	0	-	68.1	29.4	0.883	1.31	35.5
14	74	F	tongue	well	C	1	1	0	SOHND	0	0	-	43.3	23.2	0.892	1.33	23.8
15	61	M	tongue	well	C	2	2b	0	RND	0	0	-	67.0	29.3	0.874	1.37	33.0
16	60	M	tongue	well	C+R	2	0	0	RND	0	0	-	87.6	36.5	0.761	1.52	41.6
17	81	F	tongue	well	-	3	0	0	RND	0	0	-	72.7	34.5	0.732	1.69	27.9
18	55	F	tongue	well	-	3	0	0	RND	0	0	-	73.7	35.1	0.724	1.56	26.1
21	47	M	tongue	well	C	4	1	0	RND	0	0	-	57.3	30.0	0.753	1.57	29.0
22	83	M	tongue	well	R	2	0	0	RND	0	0	-	91.6	38.3	0.742	1.55	33.7
24	57	M	tongue	well	C	4	1	0	RND	0	0	-	76.7	35.6	0.724	1.68	31.0
25	45	M	tongue	well	C+R	3	2b	0	RND	0	0	-	76.9	34.3	0.784	1.61	32.1
19	61	F	tongue	moderate	-	4	0	0	RND	0	0	-	95.9	38.1	0.787	1.42	41.0
20	72	M	tongue	moderate	-	2	0	0	SOHND	0	0	-	101.4	39.9	0.765	1.49	27.6
26	82	M	tongue	moderate	C	3	1	0	SOHND	0	0	-	79.2	33.9	0.808	1.37	39.9
23	59	M	tongue	poorly	C	2	0	0	RND	0	0	-	76.3	32.7	0.858	1.43	21.8
27	79	F	upper gingiva	well	C	4	0	0	SOHND	0	0	-	131.4	44.5	0.800	1.35	30.3
28	63	F	upper gingiva	well	C	2	0	0	SOHND	0	0	-	123.3	42.3	0.834	1.25	23.0
29	53	F	upper gingiva	well	C	2	0	0	SOHND	0	0	-	48.7	27.2	0.796	1.35	25.9
31	69	F	upper gingiva	well	C	2	0	0	SOHND	0	0	-	56.3	30.3	0.741	1.75	29.9
32	84	M	upper gingiva	well	C	4	0	0	SOHND	0	0	-	55.7	29.1	0.800	1.32	22.3
30	74	M	upper gingiva	moderate	-	2	0	0	SOHND	0	0	-	53.8	28.3	0.816	1.22	23.9
33	81	F	lower gingiva	well	C	2	0	0	SOHND	0	0	-	107.4	40.3	0.791	1.31	29.7
34	74	M	lower gingiva	well	C	3	0	0	SOHND	0	0	-	72.1	33.8	0.771	1.68	22.8
35	74	F	lower gingiva	well	C	2	0	0	SOHND	0	0	-	51.7	28.2	0.784	1.45	30.7
36	81	F	lower gingiva	well	C	4	0	0	SOHND	0	0	-	65.4	31.0	0.823	1.30	30.8
37	68	M	lower gingiva	moderate	C	1	0	0	SOHND	0	0	-	61.7	30.1	0.820	1.27	29.5
38	68	F	buccal	well	-	2	0	0	SOHND	0	0	-	62.3	30.4	0.820	1.43	24.3
39	60	F	buccal	moderate	C+R	2	0	0	SOHND	0	0	-	93.9	38.6	0.772	1.68	27.5
40	63	M	mouth floor	well	C	2	1	0	SOHND	0	0	-	79.5	35.0	0.780	1.44	34.3
41	63	M	mouth floor	moderate	C+R	4	0	0	SOHND	0	0	-	80.3	35.6	0.757	1.45	38.4
42	76	M	mouth floor	poorly	C+R	4	1	0	RND	0	0	-	68.4	32.1	0.814	1.30	17.7
													73.2±19.6	32.3±4.9	0.815±0.05	1.43±0.14	31.3±6.6
																	Mean±SD
43	61	M	tongue	well	C	3	2b	0	RND	1	1	II	100.2	36.4	0.855	1.36	40.3
44	74	F	tongue	well	C	2	2c	0	RND	2c	1	I	121.2	40.6	0.830	1.47	41.8
45	71	M	tongue	well	C	2	2c	0	RND	1	1	I	102.6	36.6	0.871	1.35	40.2
46	71	F	tongue	well	C	2	0	0	RND	2b	2	II	72.0	30.0	0.891	1.29	41.1
47	58	F	tongue	well	C	4	1	0	RND	1	1	I	110.8	39.3	0.827	1.51	31.8
48	48	M	tongue	well	C	2	1	0	RND	2b	3	I+II	105.9	37.3	0.856	1.43	42.7
49	61	F	tongue	well	C	4	1	0	RND	2c	5	II	103.5	38.6	0.800	1.66	35.0
50	58	M	tongue	well	C	4	0	0	SOHND	1	1	II	71.5	30.4	0.873	1.41	35.1
51	58	F	tongue	well	C	2	0	0	RND	2b	2	I+II	110.4	38.4	0.857	1.40	37.8
52	67	M	tongue	well	C	4	1	0	RND	1	1	II	103.3	39.4	0.773	1.75	37.1
53	42	F	tongue	well	C	1	1	0	SOHND	1	1	I	140.3	44.3	0.825	1.47	46.8
54	69	F	tongue	well	C	1	0	0	RND	2b	3	II	61.2	28.1	0.866	1.42	30.7
55	69	F	tongue	well	C	3	1	0	RND	1	1	I	101.9	37.5	0.834	1.53	28.7
56	79	M	tongue	well	C	3	2b	0	RND	2b	3	II+IV	62.0	31.1	0.769	1.54	26.9
57	57	M	tongue	well	C+R	2	0	0	RND	1	1	II	67.2	32.1	0.766	1.54	36.5
58	49	M	tongue	well	C+R	2	1	0	RND	1	1	**II**	96.5	38.8	0.759	1.56	39.3
59	57	F	tongue	well	C+R	2	0	0	SMND	1	1	II	59.4	30.4	0.764	1.49	28.5
60	62	M	tongue	well	C+R	1	0	0	RND	2b	4	II+IV	67.6	32.9	0.750	1.64	30.2
61	45	M	tongue	well	C+R	4	2c	0	RND	2b	1	II	108.3	40.1	0.806	1.44	44.1
62	34	F	tongue	well	C+R	1	1	0	SOHND	1	1	II	83.1	35.5	0.798	1.47	31.2
63	62	M	tongue	well	-	1	0	0	RND	2b	4	II+IV	67.6	32.9	0.75	1.64	30.2
64	61	M	tongue	well	-	2	0	0	RND	2b	3	II	92.9	37.4	0.791	1.44	30.1
65	61	M	tongue	moderate	C	2	0	0	RND	1	1	II	86.1	35.3	0.839	1.39	27.1
66	57	M	tongue	moderate	C+R	3	2b	0	RND	2b	3	II+III	75.4	35.0	0.751	1.62	43.1
67	69	M	tongue	poorly	R	1	0	0	RND	2b	1	II	77.4	34.3	0.785	1.45	28.3
68	39	M	tongue	poorly	C+R	3	1	0	RND	2b	2	II+IV	85.0	36.8	0.757	1.44	23.1
69	44	M	upper gingiva	well	C	1	0	0	SOHND	2b	4	II+III	135.5	44.9	0.796	1.29	33.6
70	65	M	upper gingiva	well	C+R	4	0	0	RND	2b	4	II	120.1	43.9	0.745	1.39	41.2
71	81	F	upper gingiva	moderate	-	2	3	0	SOHND	2b	2	II	196.0	54.8	0.788	1.28	28.9
72	84	M	lower gingiva	well	C	2	2	0	SOHND	2b	3	II	127.0	43.0	0.831	1.30	25.0
73	64	M	lower gingiva	well	C	2	1	0	RND	1	1	II	141.3	47.2	0.764	1.28	28.7
74	68	M	lower gingiva	well	C	2	0	0	SOHND	2b	2	II	76.8	33.8	0.800	1.37	37.2
75	73	M	lower gingiva	well	C	2	1	0	SOHND	1	1	II	72.4	33.1	0.778	1.37	49.5
76	75	M	lower gingiva	well	C+R	4	0	0	SOHND	2b	2	II+III	89.5	34.1	0.789	1.35	35.7
77	59	F	lower gingiva	moderate	C	2	1	0	RND	1	1	I	157.2	48.2	0.816	1.41	31.3
78	77	M	lower gingiva	moderate	C	2	1	0	RND	1	1	II	150.1	46.8	0.831	1.31	26.3
79	76	M	lower gingiva	moderate	C	2	1	0	RND	2b	3	III	126.3	43.4	0.798	1.51	41.6
80	66	M	lower gingiva	moderate	C	4	0	0	RND	2b	2	II	119.6	42.4	0.793	1.25	35.7
81	62	M	lower gingiva	moderate	C+R	4	2	0	RND	1	1	III	93.6	37.4	0.817	1.36	22.2
82	82	F	lower gingiva	moderate	-	2	1	0	RND	1	1	III	103.5	39.6	0.795	1.33	28.5
83	53	F	buccal	well	C	4	1	0	SOHND	2b	2	II	90.7	39.6	0.696	1.42	33.6
84	65	F	buccal	well	-	2	0	0	SOHND	1	1	II	58.9	31.2	0.732	1.55	29.8
85	67	F	mouth floor	well	-	2	1	0	SOHND	1	1	II	103.2	40.1	0.777	1.65	31.4
86	54	M	mouth floor	moderate	C	2	1	0	SOHND	1	1	II	82.5	36.5	0.744	1.59	34.5
87	65	M	mouth floor	moderate	C	3	1	0	RND	1	1	II	54.8	29.1	0.770	1.38	29.9
88	55	M	mouth floor	moderate	C	2	0	0	SOHND	1	1	II	67.5	32.9	0.746	1.57	27.7
													97.6±30.2	37.6±5.7	0.799±0.04	1.44±0.11	33.9±6.5
																	Mean±SD

C: chemotherapy; FND: functional neck dissection; NAT: neoadjuvant therapy; R: radiation therapy; RND: radical neck dissection; SMND: submandibular neck dissection; SOHND: supraomohyoid neck dissection.

Histopathologic examination of the preoperative biopsy specimens identified squamous cell carcinoma in all 88 patients: well differentiated in 65, moderately differentiated in 19, and poorly differentiated in 4. Forty-six patents were pN positive, the number and level of the metastatic lymph nodes are shown in Table 2.

**Table 2:** Pathologic nodal classification and number and level of metastatic lymph nodes.

Variable	Category	Node-positive patients, n = 46
Pathologic nodal classification	1	24
	2b	20
	2c	2
No. of metastatic lymph nodes	1	27
	2	8
	3	7
	4	3
	5	1
Level of metastatic lymph nodes	I	5
	I+II	2
	II	29
	II+III	3
	II+IV	3
	III	3
	V	1

### Risks for cervical lymph node metastasis according to pN status

**Figure 3:**
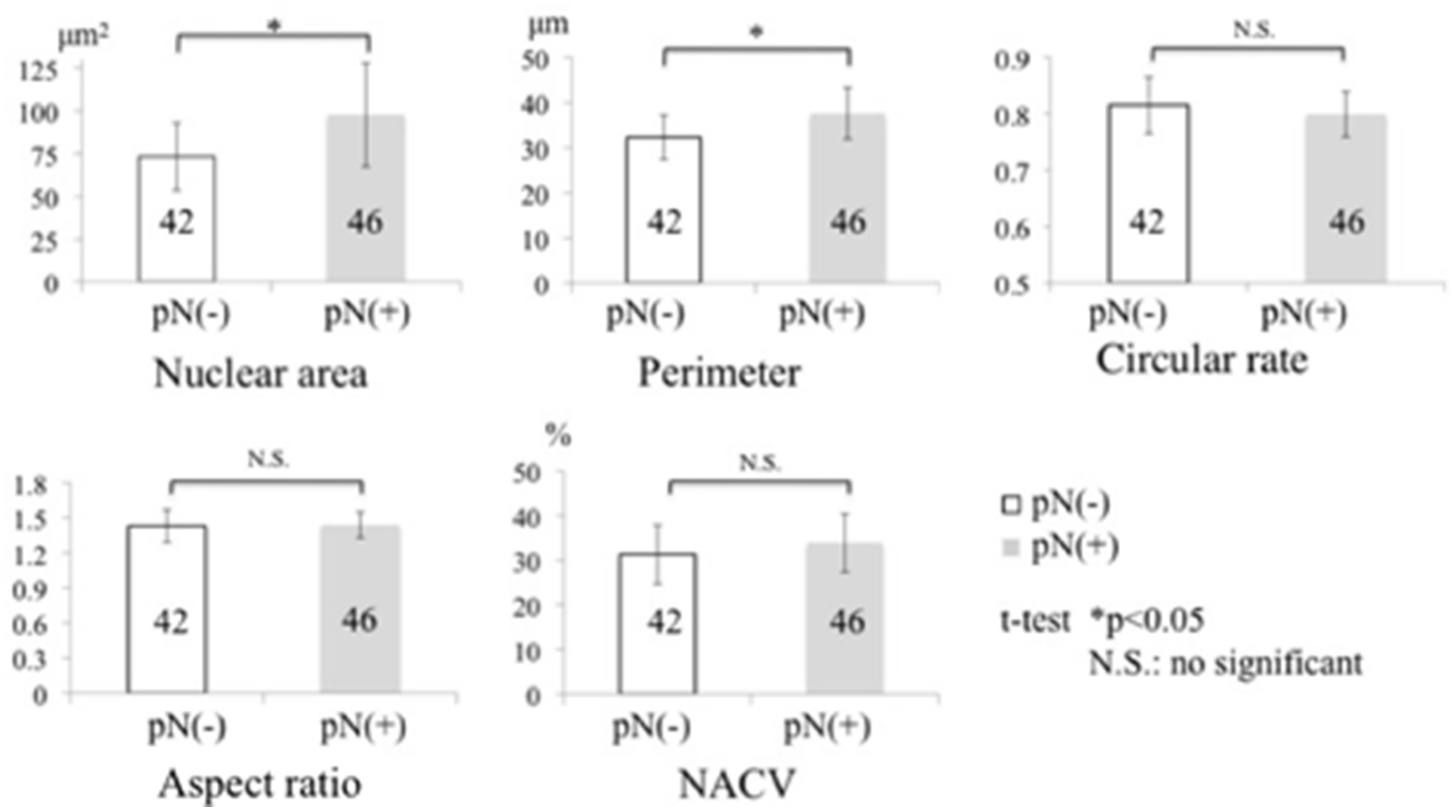
Morphometry of pathologic nodal status, pN(+) and pN(−), in all cases of OSCC.

Mean nuclear area was significantly larger in pN-positive patients than in pN-negative patients (97.6±30.2 µm^2^ and 73.2±19.6 µm^2^, respectively, *p* = 0.0226), as was mean nuclear perimeter (37.6±5.7 µm and 32.3±4.9 µm, *p* = 0.0217). However, there were no significant differences between the two groups in relation to nuclear circular rate, aspect ratio, or NACV (Fig. 3).

In univariate logistic regression analysis, the candidate risk factors associated with pN status were age (odds ratio [95% confidence interval], *p* value: 0.97 [0.93–1.00], *p* = 0.078), nuclear area (1.04 [1.02–1.07], *p*<0.001), nuclear perimeter (cutoff value, >32.7 µm) (1.23 [1.11–1.36], *p*<0.001), and nuclear circular rate (0.44 [0.18–1.09], *p* = 0.075, Table 3.

**Table 3:** Results of univariate logistic regression for the development of cervical lymph node metastasis in OSCC.

Variable		Pathologic nodal classification		Univariate logistic regression					
		Negative (n = 42)	Positive (n = 46)	Odds ratio	95% confidence interval			*p* value	Overall test
Age	(years)	66.9±11.6	62.5±11.4	0.97	0.93	-	1.00	0.078	0.078
Sex	Women	16	16	1.00					0.747
	Men	26	30	1.15	0.48	-	2.75	0.747	
Differentiation	Well	33	32	1.00					0.567
	Moderately	7	12	1.77	0.62	-	5.06	0.288	
	Poorly	2	2	1.03	0.14	-	7.77	0.976	
Tumor site	Tongue	26	26	1.00					0.544
	Lower Gingiva	5	11	2.20	0.67	-	7.22	0.194	
	Upper Gingiva	6	3	0.50	0.11	-	2.22	0.361	
	Mouth floor	3	4	1.33	0.27	-	6.56	0.723	
	Buccal mucosa	2	2	1.00	0.13	-	7.64	1.000	
Nuclear area	(µm^2^)	73.4±19.4	97.8±29.9	1.04	1.02	-	1.07	<0.001	<0.001
	≤80.3	33	15	1.00					<0.001
	>80.3	9	31	7.58	2.90	-	19.80	<0.001	
Nuclear perimeter	(µm^2^)	32.3±4.8	37.6±5.6	1.23	1.11	-	1.36	<0.001	<0.001
	≤32.7	26	8	1.00					<0.001
	>32.7	16	38	7.72	2.88	-	20.70	<0.001	
Circular rate		0.82±0.05	0.80±0.04	0.44	0.18	-	1.09	0.075	0.075
Aspect ratio		1.43±0.13	1.45±0.12	3.11	0.11	-	90.30	0.509	0.509
NACV		31.5±6.6	33.9±6.4	1.06	0.99	-	1.13	0.095	0.095

NACV: nuclear area coefficient of variation.

As a result of multivariate analysis, we derived two risk models: model 1 identified age (0.96 [0.92–1.00], *p* = 0.056) and mean nuclear area (1.05 [1.02–1.07], *p*<0.001) and model 2 identified age (1.11 [0.92–1.00], *p* = 0.075) and mean nuclear perimeter (1.23 [1.11–1.36], *p*<0.001), as risk factors associated with pN-positive status. It should be noted that primary tumor site was not associated the pN-positive status (*p* = 0.544).

## Discussion

A simple and reliable method for evaluating the preoperative risk for lymph node metastasis would be indispensable in routine clinical practice, and our approach requires no special equipment or staining technique. Several papers discuss that nuclear shape is a critical factor in the characterization of many neoplastic and non-neoplastic proliferations [8], and irregularity of the nuclear shape is one of the morphological characteristics commonly used to determine the type or degree of neoplastic transformation [34]. Recently, in the field of oral and maxillofacial surgery, several studies using morphometric analysis have evaluated the relationship between nuclear morphometry and histological grading [35]–[37], malignancies [39]–[43], and metastatic potential [14], [44]. Size and contour irregularities of the nuclei are important features in the grading of OSCC [35]–[40]. Furthermore, it was reported that the nuclear size was larger in proportion to the grade of malignancy [41], [42].

On the whole, among the quantitative morphometric parameters of the nuclei analyzed in this study, nuclear area and nuclear perimeter were significantly larger in pN-positive cases than in pN-negative cases, while the nuclear circular rate was lower in pN-positive cases but not significantly so. These results suggest that malignant nuclei become aspherical, which would be consistent with previous reports [8], [35]–[42]. Regarding the patient’s age, however, it has been reported that low age (<42 years) was associated with the development of cervical lymph node metastasis within a short time frame (≤50 days) [45]. In the present study, age<65 years was suggested to be a risk factor for cervical lymph node metastasis in OSCC.

NACV was reported to be the most feasible parameter for predicting risk for lymph node metastasis in thyroid cancer and breast cancer [29], [30]. Nuclear pleomorphism is considered the most important feature in the grading of OSCC [35]. However, in the present study, although NACV was higher in pN-positive cases it did not reach statistical significance, and thus it does not appear to be a reliable parameter for predicting risk for lymph node metastasis in OSCC.

As for the relationships we investigated between the quantitative morphometric parameters and nodal staging, nuclear area was significantly larger in pN-positive patients than in pN-negative patients. In addition, pN0 and pN2b status showed a significant difference in nuclear perimeter, nuclear circular rate, and NACV. However, the number of metastatic lymph nodes showed no significant correlation with NACV.

Briggs et al. [13] reported that nuclear morphometric measurement was useful for evaluating metastatic potential in early squamous cell carcinoma of the mouth floor. Therefore, we also examined whether the applicability of our approach would be influenced by the site of the primary OSCC tumor. However, we found no significant differences in nuclear morphometric results between pN-positive and pN-negative cases according to sites in the gingiva, buccal mucosa, mouth floor, or tongue (Table 3).

In this study, 36 of 42 patients with pN-negative disease and 40 of 46 patients with pN-positive disease received neoadjuvant chemotherapy, radiotherapy, or both. It is likely that such preoperative therapies might affect the nodal staging. While our technique of preoperative nuclear morphometry using biopsy specimens appears to be applicable to help decide whether the lymph nodes harbor metastases or not, retrospective studies in general are insufficient to discuss a cause-effect relationship for the metastasis from evaluating the lymph node profiles alone (e.g., nodal staging and number of nodes). It is essential, therefore, to carry out a prospective study to verify the applicability of our method for predicting cervical lymph node metastasis.

Future studies also need to address the applicability of clinical and biological marker analyses to accurately evaluate metastatic potential in OSCC preoperatively. Recently, the nuclear factor kappa B was reported to be a key protein in multi-step carcinogenesis, lymph node metastasis, and prognosis of oral, head, and neck squamous cell carcinoma [44], [46]. The expression of a combination of nuclear factor kappa B or other markers should be examined further.

In conclusion, our method of preoperative nuclear morphometry may contribute valuable information to evaluations of the risk for lymph node metastasis in OSCC.

## References

[1] Kim KY, Cha IH (2011) A novel algorithm for lymph node status prediction of oral cancer before surgery. Oral Oncol 47:1069–1073. 21840750 10.1016/j.oraloncology.2011.07.017

[2] Lim YC, Koo BS, Lee JS, Choi EC (2006) Level V lymph node dissection in oral and oropharyngeal carcinoma patients with clinically node-positive neck: Is it absolutely necessary? Laryngoscope 116:1232–1235. 16826066 10.1097/01.mlg.0000224363.04459.8b

[3] Montes DM, Carlson ER, Fernandes R, Ghali GE, Lubek J, et al (2011) Oral maxillary squamous carcinoma: an indication for neck dissection in the clinically negative neck. Head Neck 33:1581–1585. 21990223 10.1002/hed.21631

[4] Liao CT, Hsueh C, Lee LY, Lin CY, Fan KH, et al (2012) Neck dissection field and lymph node density predict prognosis in patients with oral cavity cancer and pathological node metastases treated with adjuvant therapy. Oral Oncol 48:329–336. 22104249 10.1016/j.oraloncology.2011.10.017

[5] Hoch S, Fasunla J, Eivazi B, Werner JA, Teymoortash A. (2012) Delayed lymph node metastases after elective neck dissection in patients with oral and oropharyngeal cancer and pN0 neck. Am J Otolaryngol 33:505–509. 22218151 10.1016/j.amjoto.2011.11.005

[6] Kang CJ, Liao CT, Hsueh C, Lee LY, Lin CY, et al (2011) Outcome analysis of patients with well-differentiated oral cavity squamous cell carcinoma. Oral Oncol 47:1085–1091. 21840751 10.1016/j.oraloncology.2011.07.018

[7] van den Brekel MW, van der Waal I, Meijer CJ, Freeman JL, Castelijns JA, et al (1996) The incidence of micrometastases in neck dissection specimens obtained from elective neck dissections. Laryngoscope 106:987–991. 8699914 10.1097/00005537-199608000-00014

[8] Sekine J, Uehara M, Hideshima K, Irie A, Inokuchi T. (2011) Predictability of lymph node metastases by preoperative nuclear morphometry in squamous cell carcinoma of the tongue. Cancer Detect Prev 27:427–433. 10.1016/j.cdp.2003.09.001 14642550

[9] Flach GB, Tenhagen M, de Bree R, Brakenhoff RH, van der Waal I, et al (2013) Outcome of patients with early stage oral cancer managed by an observation strategy towards the N0 neck using ultrasound guided fine needle aspiration cytology: No survival difference as compared to elective neck dissection. Oral Oncol 49:157–164. 22967965 10.1016/j.oraloncology.2012.08.006

[10] Nathanson SD (2003) Insights into the mechanisms of lymph node metastasis. Cancer 98:413–423. 12872364 10.1002/cncr.11464

[11] Crile G (1906) Excision of cancer of the head and neck with special reference to the plan of dissection based upon one hundred thirty-two operations. J Am Med Assoc 47:1780–1786. 10.1001/jama.258.22.3286 3316722

[12] MacCarthy D, Flint SR, Healy C, Stassen LF (2011) Oral and neck examination for early detection of oral cancer – a practical guide. J Ir Dent Assoc 57:195–199. 21922994

[13] Briggs RJ, Pienta KJ, Hruban RH, Richtsmeier WJ (1992) Nuclear morphometry for prediction of metastatic potential in early squamous cell carcinoma of the floor of the mouth. Arch Otolaryngol Head Neck Surg 118:531–533. 1571129 10.1001/archotol.1992.01880050085020

[14] Vázquez-Mahía I, Seoane J, Varela-Centelles P, Tomás I, Álvarez García A, et al (2012) Predictors for tumor recurrence after primary definitive surgery for oral cancer. J Oral Maxillofac Surg 70:1724–1732. 21940087 10.1016/j.joms.2011.06.228

[15] Okura M, Kagamiuchi H, Tominaga G, Iida S, Fukuda Y, et al (2005) Morphological changes of regional lymph node in squamous cell carcinoma of the oral cavity. J Oral Pathol Med 34:214–219. 15752256 10.1111/j.1600-0714.2005.00304.x

[16] Chone CT, Aniteli MB, Magalhães RS, Freitas LL, Altemani A, et al (2013) Impact of immunohistochemistry in sentinel lymph node biopsy in head and neck cancer. Eur Arch Otorhinolaryngol 270:313–317. 22566179 10.1007/s00405-012-2032-5

[17] Lim SC, Zhang S, Ishii G, Endoh Y, Kodama K, et al (2004) Predictive markers for late cervical metastasis in stage I and II invasive squamous cell carcinoma of the oral tongue. Clin Cancer Res 10:166–172. 14734465 10.1158/1078-0432.ccr-0533-3

[18] Kawano K, Yanagisawa S (2006) Predictive value of laminin-5 and membrane type 1-matrix metalloproteinase expression for cervical lymph node metastasis in T1 and T2 squamous cell carcinomas of the tongue and floor of the mouth. Head Neck 28:525–533. 16619276 10.1002/hed.20349

[19] Harada H, Omura K, Nakajima Y, Hasegawa S, Mogi S (2006) Cyclin B1 is useful to predict occult cervical lymph node metastases in tongue carcinoma. J Exp Cancer Res 25:351–356. 17167975

[20] Ferris RL, Kraus DH (2012) Sentinel lymph node biopsy versus selective neck dissection for detection of metastatic oral squamous cell carcinoma. Clin Exp Metastasis 29:693–698. 22711338 10.1007/s10585-012-9492-2

[21] Stoeckli SJ, Broglie MA (2012) Sentinel node biopsy for early oral carcinoma. Curr Opin Otolaryngol Head Neck Surg 20:103–108. 22202232 10.1097/MOO.0b013e32834ef6d3

[22] van der VorstJR, Schaafsma BE, Verbeek FP, Keereweer S, Jansen JC, et al (2012) Near-infrared fluorescence sentinel lymph node mapping of the oral cavity in head and neck cancer patients. Oral Oncol 49:15–19. 22939692 10.1016/j.oraloncology.2012.07.017 PMC3608510

[23] Matsuzuka T, Kano M, Ogawa H, Miura T, Tada Y, et al (2008) Sentinel node mapping for node positive oral cancer: Potential to predict multiple metastasis. Laryngoscope 118:646–649. 18176344 10.1097/MLG.0b013e3181613aa6

[24] Manola M, Aversa C, Moscillo L, Villano S, Pavone E, et al (2011) Status of level IIb lymph nodes of the neck in squamous cell carcinoma of the oral tongue in patients who underwent modified radical neck dissection and lymph node sentinel biopsy. Acta Otorhinolaryngol Ital 31:130–134. 22058590 PMC3185818

[25] Iñarra Unzurrunzaga E, Gorriño Angulo M, Vidales Arechaga L, Aguirre Larracoechea U, Iriondo BedialaunetaJR. (2011) Predictive Ability of the CT to evaluate cervical lymph nodes in head and neck tumours. Acta Otorrinolaringol Esp 62:443–447. 21958675 10.1016/j.otorri.2011.06.004

[26] Keereweer S, Kerrebijn JD, Mol IM, Mieog JS, Van Driel PB, et al (2012) Optical imaging of oral squamous cell carcinoma and cervical lymph node metastasis. Head Neck 34:1002–1008. 21987435 10.1002/hed.21861

[27] Ishibashi N, Yamagata K, Sasaki H, Seto K, Shinya Y, et al (2012) Real-time tissue elastography for the diagnosis of lymph node metastasis in oral squamous cell carcinoma. Ultrasound Med Biol 38:389–395. 22266228 10.1016/j.ultrasmedbio.2011.12.004

[28] Jerjes W, Upile T, Radhi H, Petrie A, Abiola J, et al (2012) cTNM vs. pTNM: the effect of not applying ultrasonography in the identification of cervical nodal disease. Head Neck Oncol 4:5. 22410339 10.1186/1758-3284-4-5 PMC3351374

[29] Suzuki M, Oshida M, Nagashima T, Hashimoto H, Yagata H, et al (2001) Quantitative morphometric analysis of fine needle aspiration of breast carcinoma. Breast Cancer 8:138–145. 11342987 10.1007/BF02967493

[30] Nagashima T, Suzuki M, Yagata H, Hashimoto H, Shishikura T, et al (2000) Cytomorphometric differentiation of intraductal proliferative breast lesions. Breast Cancer 7:43–47. 11029770 10.1007/BF02967187

[31] Werner JA, Dunne AA, Myers JN. (2003) Functional anatomy of the lymphatic drainage system of the upper aerodigestive tract and its role in metastasis of squamous cell carcinoma. Head Neck 25:322–332. 12658737 10.1002/hed.10257

[32] Shah JP, Candela FC, Poddar AK. (1990) The patterns of cervical lymph node metastases from squamous carcinoma of the oral cavity. Cancer 66:109–113. 2354399 10.1002/1097-0142(19900701)66:1<109::aid-cncr2820660120>3.0.co;2-a

[33] Loré JM. (1999) An atlas of head and neck surgery, 3rd ed. Philadelphia: Saunders. 645–669 p.

[34] Wydner KS, Godyn JJ, Lee ML, Sciorra LJ. (1991) A new approach to the computer-assisted quantitative analysis of nuclear shape. Mod Pathol 4:154–160. 2047379

[35] Giardina C, Serio G, Caniglia DM, Lettini T, Ricco R, et al (1994) Nuclear morphology and histological grading of oral squamous cell carcinoma (OSCC). A morphometric study. Boll Soc Ital Biol Sper 70:271–279. 7702831

[36] Giardina C, Caniglia DM, D’Aprile M, Lettini T, Serio G, et al (1996) Nuclear morphometry in squamous cell carcionoma (SCC) of the tongue. Eur J Cancer B Oral Oncol 32B:91–96. 8736170 10.1016/0964-1955(95)00062-3

[37] Nandini DB, Subramanyam RV. (2011) Nuclear features in oral squamous cell carcinoma: A computer-assisted microscopic study. J Oral Maxillofac Pathol 15:177–181. 22529576 10.4103/0973-029X.84488 PMC3329696

[38] White FH, Jin Y, Yang L. (1997) An evaluation of the role of nuclear cytoplasmic ratios and nuclear volume densities as diagnostic indicators in metaplastic, dysplastic and neoplastic lesions of the human cheek. Histol Histopathol 12:69–77. 9046045

[39] Pektas ZO, Keskin A, Günhan O, Karslioğlu Y. (2006) Evaluation of nuclear morphometry and DNA ploidy status for detection of malignant and premalignant oral lesions: quantitative cytologic assessment and review of methods for cytomorphometric measurements. J Oral Maxillofac Surg 64:628–635. 16546642 10.1016/j.joms.2005.12.010

[40] Smitha T, Sharada P, Girish H. (2011) Morphometry of the basal cell layer of oral leukoplakia and oral squamous cell carcinoma using computer-aided image analysis. J Oral Maxillofac Pathol 15:26–33. 21731274 10.4103/0973-029X.80034 PMC3125652

[41] Martano M, Damiano S, Restucci B, Paciello O, Russo V, et al (2006) Nuclear morphometry in canine acanthomatous ameloblastomas and squamous cell carcinomas. Eur J Histochem 50:125–130. 16864123

[42] van der Wal N, Baak JP, Schipper NW, van der Waal I. (1989) Morphometric study of pseudoepitheliomatous hyperplasia in granular cell tumors of the tongue. J Oral Pathol Med 18:8–10. 2545872 10.1111/j.1600-0714.1989.tb00723.x

[43] Natarajan S, Mahajan S, Boaz K, George T. (2010) Prediction of lymph node metastases by preoperative nuclear morphometry in oral squamous cell carcinoma: a comparative image analysis study. Indian J Cancer 47:406–411. 21131754 10.4103/0019-509X.73580

[44] Yan M, Xu Q, Zhang P, Zhou XJ, Zhang ZY, et al (2010) Correlation of NF-?B signal pathway with tumor metastases of human head and neck squamous cell carcinoma. BMC Cancer 10:437 doi:–––10.1186/1471–2407–10–437. 20716363 10.1186/1471-2407-10-437 PMC2931490

[45] Keiko U-N, Hitoshi S, Ryoichi Y, Masahiko M, Hiroshi W, et al (2013) Cervical lymph node metastasis from early-stage squamous cell carcinoma of the tongue. Acta Oto-Laryngologica 133:544–551. 23350600 10.3109/00016489.2012.748988

[46] Nariai Y, Mishima K, Yoshimura Y, Sekine J. (2011) FAP-1 and NF-?B expression in oral squamous cell carcinoma as potential markers for chemo-radio sensitivity and prognosis. Int J Oral Maxillofac Surg 40:419–426. 21176871 10.1016/j.ijom.2010.10.020

